# Simultaneous Versus Staged Bilateral Hip Arthroscopy for Femoroacetabular Impingement: Minimum 2-Year Outcomes With a Unilateral Control Group

**DOI:** 10.1177/03635465251328605

**Published:** 2025-04-10

**Authors:** Gen Lin Foo, Matthew J. Brick, Catherine J. Bacon

**Affiliations:** * Orthosports North Harbour Ltd, Auckland, New Zealand; † Apex Sports Clinic, Singapore; ‡ School of Nursing, Faculty of Medical and Health Sciences, University of Auckland, Auckland, New Zealand; Investigation performed at Orthosports North Harbour Ltd, Auckland, New Zealand

**Keywords:** femoroacetabular impingement, orthopaedic procedures, hip arthroscopic surgery, patient outcome assessment, revision surgery, arthroplasty, hip replacement

## Abstract

**Background:**

One-fifth of patients with femoroacetabular impingement (FAI) have bilateral symptoms. Performing bilateral hip arthroscopy on the same day minimizes the overall rehabilitation period compared with staged bilateral surgery, but most studies of outcomes from simultaneous surgery are in small cohorts.

**Purpose/Hypothesis:**

The purpose was to compare clinical outcome and revision rates between patients undergoing simultaneous bilateral, staged bilateral, and unilateral arthroscopic surgery for FAI from a large single-surgeon cohort. It was hypothesized that there would be no between-group differences.

**Study Design:**

Cohort study; Level of evidence, 3.

**Methods:**

Simultaneous bilateral, staged bilateral, and unilateral primary hip arthroscopy procedures between June 2005 and December 2020 were identified. Patient-reported outcome measures including the 12-item International Hip Outcome Tool (iHOT-12) score, Non-Arthritic Hip (NAH) score, and Hip Disability and Osteoarthritis Outcome Score (HOOS) were collected preoperatively and at regular intervals postoperatively. Two-year follow-up scores were analyzed if they were available, or later follow-ups if they were not. Subsequent surgery rates were recorded utilizing a compulsory national joint replacement registry.

**Results:**

A total of 196 patients (392 hips) in the simultaneous bilateral and 111 patients (222 hips) in the staged bilateral groups were compared with 1529 patients in the unilateral group. The median duration between staged surgeries was 62 days (range, 14-350 days), and demographic characteristics were similar for those having simultaneous and staged procedures. Two-year minimum postoperative scores in all 3 groups were significantly improved from preoperative scores (*P* < .001). Improvements were similar between groups for all scores apart from HOOS-Sports (*P* = .03) and HOOS–Quality of Life (*P* = .03), which improved less in the staged compared with the other 2 groups, and for HOOS–Quality of Life only, which attained a lower follow-up score for staged (mean, 63.1 ± 24.7) compared with simultaneous (mean, 69.8 ± 22.7) procedures (*P* = .04 for post hoc pairwise comparison). For the iHOT-12 score (*P* = .04), HOOS-Sports (*P* = .02), and HOOS-QoL (*P* = .02), a lower proportion of patients receiving staged compared with other procedures achieved minimally important clinical differences. No differences between groups in revision or arthroplasty conversion rates adjusted for follow-up time were observed.

**Conclusion:**

Patients undergoing simultaneous bilateral arthroscopy for FAI achieved similar 2-year follow-up outcomes compared with staged and unilateral arthroscopy and performed better than the staged group in Sports and Quality of Life subscales of the HOOS.

Femoroacetabular impingement (FAI) was first described by Ganz et al^10^ in 2003 as a clinical entity that can predispose patients to early-onset osteoarthritis.^5^ There are 2 distinct types of FAI morphology, cam type with a prominent femoral head-neck junction and pincer type secondary to acetabular overcoverage.^11^ Impingement leads to chondrolabral injuries, which adversely affect hip joint kinematics, damage the labral suction seal, and increase contact and shear forces leading to accelerated degeneration.

The surgical treatment for FAI can be categorized into open procedures (eg, surgical hip dislocation and osteoplasty, reverse periacetabular osteotomy) and arthroscopic surgery. Arthroscopic hip surgery has become well established following early indications of favorable improvements in patient-reported outcome measures (PROMs) and fast return to high-level sports.^2,16,26,27^ Later studies demonstrated greater improvements in PROMs with hip arthroscopy for FAI compared with open procedures^22^ and compared with nonoperative management.^12,25^

Patients with FAI often demonstrate similar radiographic features in both hips even when symptoms are present in only 1 hip. Allen et al^1^ reported that 78% of patients with FAI arthroscopy had bilateral cam morphology, of whom only one-quarter had bilateral symptoms. In contrast, a more recent study identified bilateral radiographic signs of FAI in only 31% of patients evaluated for FAI surgery, of whom 52% had bilateral symptoms initially but a further 35% became symptomatic after a mean of 2 years.^4^ In both cases, 20% to 25% of patients with FAI surgery have eventual bilateral symptoms, consistent with another study reporting that patients having a second hip operated on comprised a similar proportion of total surgical load.^13^

One debate on the optimal treatment for this group of patients is centered on whether they should be offered simultaneous or staged surgery and, if staged, the ideal interval between the index and contralateral surgery. Simultaneous bilateral surgery can reduce the overall rehabilitation period, allowing patients to return to sport or work faster.^20^ This translates to economic savings through both less time off work and reduced health care costs (single admission, anesthesia, surgery, expensive single-use instruments, and rehabilitation). However, there are concerns about potentially higher complication rates from the longer surgical and traction times required as well as the possible compromise of effective rehabilitation. To execute an expedient simultaneous bilateral surgery, there is also a need for an experienced surgeon and team.

The first paper demonstrating the safety and efficacy of simultaneous bilateral arthroscopic FAI surgery was published in 2014 by our group and reported outcomes from 26 simultaneous bilateral, 20 staged bilateral, and 33 unilateral patients.^20^ Although later papers have shown encouraging results for simultaneous bilateral versus unilateral surgery,^18^ or bilateral versus unilateral surgery,^3,6,15^ most involve small numbers of patients and have modest statistical power for detecting any difference in procedures. Results from a 2020 systematic review show similar efficacy of bilateral compared with unilateral hip arthroscopy with no increased risk of complications.^9^ Collating results from the 6 available studies introduced different surgeons and surgical groups as an additional source of variability when comparing outcomes between groups. In addition, the total sample size is still relatively small—67 patients in the simultaneous bilateral group, 144 patients in the staged bilateral group, and 511 patients in the unilateral group—and insufficient to establish if simultaneous bilateral surgery is comparable.^9^

With a decade having passed since the first simultaneous bilateral FAI surgery paper, it is imperative to revisit this question with larger numbers and extended follow-up to ensure there are no unexpected negative consequences of this approach. The purpose of this study was to compare the clinical outcomes of patients who have undergone simultaneous bilateral surgery with those of patients who have undergone staged bilateral surgery and unilateral surgery in a large single-surgeon (M.J.B.) cohort with a 2-year minimum follow-up. We hypothesized that there would be no difference in clinical outcomes between these 3 groups.

## Methods

### Patient Characteristics

Patients who underwent simultaneous bilateral, staged bilateral, and unilateral primary hip arthroscopy for FAI between June 2005 and December 2020 were identified from our surgical database. Data are prospectively collected in the database as part of an ongoing observational study approved by Northern A Health and Disability Ethics Committee, New Zealand Ministry of Health (17/NTA/269).

The inclusion criteria included hip pain that has failed nonoperative management of at least 6 months with positive clinical and radiographical signs of FAI. Patients with hip osteoarthritis of Tönnis grade 2 and above, inflammatory arthritis, hip dysplasia (lateral center-edge angle [LCEA] <20°), and previous hip surgery were excluded from the study.

Patient demographic data, including age, sex, body mass index (BMI) from height and weight, and basic surgical characteristics, were extracted from the surgeon’s database. The primary outcome was 2-year change in the 12-item International Hip Outcome Tool (iHOT-12) score. Secondary outcomes included other PROMs and subsequent ipsilateral hip surgery. Other PROMs were the Non-Arthritic Hip (NAH) score and the 5 subscales of the Hip Disability and Osteoarthritis Outcome Score (HOOS): Pain, other Symptoms, function in Activities of Daily Living (ADL), function in sports and recreation (Sports), and hip-related Quality of Life (QoL). These were collected preoperatively and at 2, 5, and 10 years. The PROMs were collected with the use of an online scoring system, Socrates Orthopaedic Outcomes Software. Traditional paper-based mail options were also available when required. The occurrence of revision surgery was documented, and conversion to total hip arthroplasty (THA) was recorded by matching a unique national health identifier number with a compulsory national joint replacement registry.

### Surgical Planning and Technique

The decision to offer simultaneous surgery to patients with bilateral symptomatic FAI was based on a range of factors. Patients with cartilage injury (assessed on preoperative radiographs or magnetic resonance imaging scans) that will likely require a cartilage procedure (eg, microfracture), borderline hip dysplasia (defined as an LCEA of 20°-25°), or microinstability are not typically offered simultaneous surgery due to the likely need for the standard 6 weeks of protected weightbearing for these groups. Patients who were deemed suitable for simultaneous bilateral surgery were offered both simultaneous and staged options after a discussion of the advantages and disadvantages of both. Simultaneous surgery offers the benefit of having just 1 surgery and a shorter overall postoperative rehabilitation because there is only 1 rehabilitation period. This allows a faster return to sports and/or work. The risk of increased traction-related complications was relayed to the patient. They were also informed about the surgical algorithm for simultaneous surgery, which might require conversion to a staged approach.

For patients who decided on the simultaneous option, the less symptomatic hip was operated on first. The logic of this approach is that if the less symptomatic hip unexpectedly requires microfracture, the previous agreement is that treatment will switch to a staged approach. If the first hip is suitable for full weightbearing, then it becomes immaterial whether the worse hip requires microfracture. Thus, 1 weightbearing hip is always assured. We do not advocate simultaneous surgery that leads to the need for protected weightbearing of both hips with a wheelchair, as this would be highly inconvenient to patients and negates the upside of faster rehabilitation.

Surgeries were performed with the patient under general anesthesia in the lateral position using the McCarthy hip distractor. Distraction was applied with the operated hip in about 10° of flexion, 20° of abduction, and neutral rotation. With adequate traction applied to access the hip joint safely, the central compartment was first accessed using the mid-trochanteric portal. With dry arthroscopic guidance using a 30° arthroscope, the anterior portal was made under direct visualization. Arthroscopic fluid was pumped with a pressure of 40 mm Hg. Beaver blades and radiofrequency probes were utilized to perform interportal capsulotomies. Diagnostic arthroscopy was performed to assess for any central compartment pathology.

For pincer FAI, acetabuloplasty was performed with a 4.5-mm bur guided visually and confirmed by fluoroscopy. Labral repair and cartilage procedures (debridement or microfracture, depending on the extent of cartilage injury) were performed. A distal anterolateral accessory portal was created depending on the need to optimize the placement of anchors for the labral repair.

Once the central compartment work was completed, traction was released and the labrum was assessed to ensure that it formed a fluid seal around the femoral head. For cam FAI, femoroplasty was performed with a 5.5-mm bur. We utilized fluoroscopy to guide the femoroplasty to achieve a “light bulb” femoral head shape with a smooth transition from the concave surface of the femoral neck to the convex surface of the femoral head. Care was taken to prevent extensive medial overresection, which can cause a “shark bite” appearance with loss of the hip’s suction seal integrity. Dynamic impingement tests were performed to check for any remnant impingement with hip flexion to 120° and internal rotation to 30° Final fluoroscopic checks, which include the anterior-posterior view and Dunn view (at 45° and 90°), were performed to confirm adequate resection. The capsule was routinely closed with at least 3 interrupted absorbable Vicryl No. 2 sutures.

After completion of the first hip, patients receiving simultaneous surgery were repositioned for the contralateral hip. Surgical instruments were retained and kept sterile throughout. The contralateral hip was prepared and draped accordingly, and the surgery was performed in the same sequence and manner as the initial hip.

Patients were kept overnight and discharged the next day per routine practice for patients undergoing all hip arthroscopy. Weightbearing as tolerated, with crutches, was prescribed for 2 to 3 weeks for patients who underwent simultaneous surgery. For those in the unilateral and staged groups, the prescription was for partial weightbearing on the operated side with crutches for 2 weeks. Patients with hyperlaxity required 4 weeks, while those who had borderline hip dysplasia and those who underwent a microfracture procedure required 6 weeks of partial weightbearing. Immediate range of motion exercises, which included riding on a stationary bicycle (without resistance), were encouraged. Core and upper limb exercises were prescribed 2 weeks after the surgery. Lower limb resistance training was started about 4 to 6 weeks after surgery. Patients were followed up in the clinic at 1 week, 3 months, and 1 year after surgery.

### Statistical Analysis

Sample size was based on a consecutive series of eligible surgeries and comprised all available within the specified time frame. With the numbers available, we calculated, using G*Power Version 3.1.97,^8^ 80% statistical power to detect a difference in change in iHOT-12 score between the simultaneous and staged groups of 8.5 units, based on a calculated effect size of 0.34, standard deviation in change in iHOT-12 score of 25 units, and alpha of .05.

Age, sex, and BMI differences between the 3 groups were checked using 1-way analysis of variance (ANOVA). Two-year follow-up patient-reported outcome scores were analyzed; later follow-up scores were substituted when 2-year scores were missing. Changes in outcome scores from baseline to the 2-year minimum follow-up were checked for large violations of assumptions of normality by visual inspection of their distribution and analysis of skewness and kurtosis z-scores for those falling outside 95% confidence limits.

Two-way mixed ANOVA (group × time) was used to compare the change from preoperative to 2-year minimum follow-up scores between groups, with the interaction’s statistical significance reported. A preplanned 1-way ANOVA for between-group final scores was undertaken when the 2-way ANOVA was statistically significant, with Bonferroni post hoc group pairwise comparisons applied. Finally, we reanalyzed pre- and postoperative repeated iHOT-12 measures, this time including potential confounding variables as covariates in the 2-way mixed ANOVA models. Minimal clinically important change (MCIC) was calculated as half the standard deviation of preoperative scores, using the method of Norman et al.^21^ The chi-square test was used to compare the rate of revision surgery and conversion to THA between the 3 groups. Throughout, a *P* value <.05 was considered statistically significant. Statistical analyses were performed with IBM SPSS Statistics for Windows Version 29.0 (IBM Corp).

## Results

**Figure 1.**
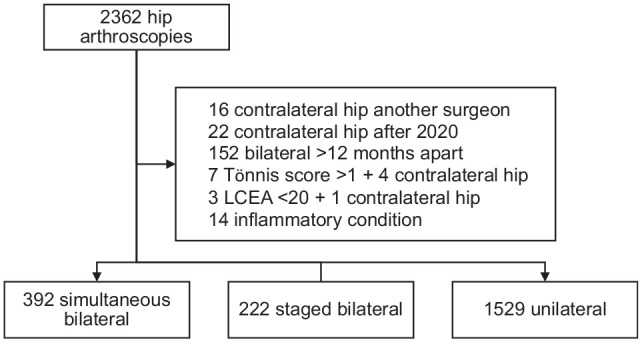
Patient selection flowchart. LCEA, lateral center-edge angle.

From 2362 primary hip arthroscopies identified from the surgeon’s database to December 2020, 219 were removed because they did not meet exclusion criteria, as shown in Figure 1. In addition to study exclusion criteria, staged bilateral surgeries for which the primary surgery for 1 hip was with another surgeon or was after 2020, or where surgeries were completed >12 months apart, were excluded from analysis. A total of 2143 arthroscopies remained: 196 patients (392 hips) in the simultaneous bilateral group, 111 patients (222 hips) in the staged bilateral group, and 1529 patients and hips in the unilateral group. The mean interval between surgeries for the staged bilateral group was 104 ± 94 days (median, 62 days; range, 14-350 days).

The patients in the simultaneous and staged groups were similar in terms of age, sex, BMI, LCEA, femoral head-neck offset, and proportion who had a labral tear; however, those in the unilateral group were older, more likely to be female, had a greater femoral head-neck offset than both other groups, and had a lower LCEA than the simultaneous group (*P* < .001 for all) (Table 1). As expected from surgical selection, a very low proportion of the simultaneous group had borderline hip dysplasia (LCEA 20°-25°), and consequently showed the highest mean LCEA compared with other groups (Table 1). Those in the unilateral group were more likely to report sudden onset of symptoms (*P* < .001, chi-square) (Table 1). As expected, those in the simultaneous group were less likely than those in the staged group to have a Tönnis grade of 1 (as opposed to 0) (*P* = .03, chi-square) but were unexpectedly less likely than both other groups to have no chondral damage and more likely than the staged group to have 0 to 1 microfracture holes drilled, but less likely than the staged group to have ≥5 microfracture holes (Table 1).

**Table 1:** Patient and Surgery Characteristics^*a*^

	Simultaneous	Staged	Unilateral	*P*
No. of hips	392	222	1529	
Age, y	29.8 ± 11.1	31.0 ± 11.2	37.0 ± 12.1	<.001
BMI, kg/m^2^	24.9 ± 3.5	25.2 ± 4.1	25.6 ± 4.6	.1
LCEA, deg	35.3 ± 5.3	34.4 ± 7.4	33.8 ± 6.1	<.001
20°-25°	8 (2.0)	29 (13.2)	129 (8.6)	<.001
FHN offset, mm	3.6 ± 2.5	4.1 ± 3.5	5.7 ± 3.0	<.001
Sex, male/female	260/132 (66.3/33.7)	146/76 (65.8/34.2)	629/900 (41.1/58.9)	<.001
Labral tear	344 (87.8)	192 (86.5)	1318 (86.2)	.8
Symptom onset, sudden/gradual	146/204 (41.7/58.3)	87/88 (49.7/50.3)	1026/438 (70.1/29.9)	<.001
Tönnis grade 1	14 (3.6)	18 (8.1)	67 (4.4)	.03
Rim lesion extent^*b*^				<.001
None/normal	37 (9.4)	57 (25.7)	425 (27.9)	
Wave sign	74 (18.9)	26 (11.7)	300 (19.7)	
Early delamination	92 (23.5)	39 (17.6)	371 (24.3)	
Delamination <1/3	123 (31.4)	46 (20.7)	250 (16.4)	
Delamination 1/3-2/3	66 (16.8)	54 (24.3)	178 (11.7)	
Microfracture holes				<.001
None	267 (68.1)	145 (65.3)	1173 (76.7)	
1	17 (4.3)	4 (1.8)	38 (2.5)	
2	22 (5.6)	14 (6.3)	72 (4.7)	
3	34 (8.7)	21 (9.5)	101 (6.6)	
4	31 (7.9)	13 (5.9)	73 (4.8)	
5	14 (3.6)	12 (5.4)	40 (2.6)	
>5	7 (1.8)	13 (5.9)	32 (2.1)	

a Data are per surgery and are presented as frequency (% of nonmissing) with chi-square *P* value reported or mean ± SD with analysis of variance *P* value reported. BMI, body mass index; FHN, femoral head-neck; LCEA, lateral center-edge angle.

b Modified Konan scale.^14^

**Figure 2.**
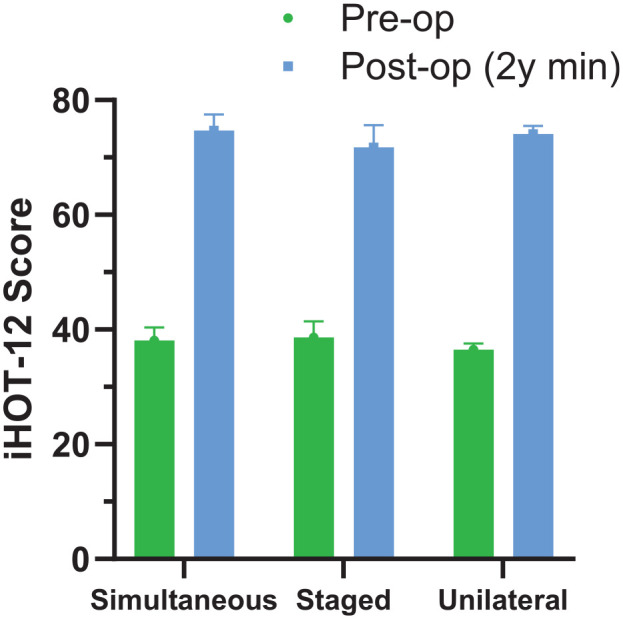
Preoperative and 2-year minimum postoperative12-item International Hip Outcome Tool (iHOT-12) scores (mean ± SD).

The mean 2-year minimum follow-up response rates across all PROMs for simultaneous bilateral, staged bilateral, and unilateral surgeries were 69.5%, 68.5% and 70.2%, respectively. The mean response rates for change scores (including patients who had both preoperative and follow-up scores available) were 54.7%, 50.1%, and 57.9% for simultaneous, staged, and unilateral surgeries, respectively. Most changes in scores met assumptions of normality; of 42 skewness and kurtosis statistics for each of the 3 groups, 12 (29%) fell outside 95% confidence limits and parametric test statistics are reported. Scores in all groups improved pre- to postoperatively (*P* < .001 for all ANOVA time effects) (Table 2). Smaller increases were noted for staged surgeries than for simultaneous or unilateral procedures for HOOS-Sports and HOOS-QoL subscales (*P* = .025 and *P* = .034, respectively, for time × group interaction effect) (Table 2). In 1-way ANOVA of follow-up scores for these 2 subscales, only QoL differed between groups (*P* = .044), being greater for simultaneous compared with staged surgeries (*P* = .042). There were no group differences for iHOT-12 total scores or their pre- to postoperative changes (Figure 2). Because there were other differences between groups, we reran repeated-measures ANOVA models for iHOT-12 total score including the following covariates: age, sex, presence of borderline dysplasia (LCEA between 20° and 25°), symptom onset (sudden vs gradual), any Tönnis grade, the extent of acetabular rim lesion, and number of microfracture holes. With these covariates included in the model, between-group differences in iHOT-12 pre- to postoperative change remained nonsignificant (*P* = .282). Removing the unilateral group from analysis, to compare only bilateral simultaneous and staged procedures, also showed no significant differences in iHOT-12 change between these 2 groups (*P* = .079 without covariates; *P* = .105 with covariates included).

**Table 2:** Preoperative and 2-Year Minimum Postoperative Patient-Reported Outcomes^*a*^

	Simultaneous		Staged		Unilateral		*P* ^*b*^
	Preoperative	2-y Minimum	Preoperative	2-y Minimum	Preoperative	2-y Minimum	
iHOT-12 score	37.2 ± 18.7	75.0 ± 22.4	38.5 ± 17.5	70.8 ± 24.8	37.3 ± 18.7	74.2 ± 23.9	.176
NAH score	61.7 ± 17.0	86.1 ± 14.2	60.3 ± 17.3	83.3 ± 17.5	60.4 ± 17.9	84.6 ± 15.3	.786
HOOS-Symptoms	53.3 ± 19.4	77.5 ± 18.5	57.2 ± 18.7	76.5 ± 20.8	58.2 ± 19.0	79.5 ± 17.0	.080
HOOS-Pain	59.7 ± 18.8	85.2 ± 16.5	60.9 ± 18.5	81.7 ± 19.9	58.6 ± 18.8	83.4 ± 16.7	.107
HOOS-ADL	68.8 ± 19.5	90.5 ± 14.3	68.2 ± 20.2	87.3 ± 19.4	67.5 ± 20.5	89.6 ± 14.8	.310
HOOS-Sports	43.2 ± 22.6	78.5 ± 20.3	47.9 ± 23.8	74.5 ± 24.6	44.6 ± 23.5	77.2 ± 21.7	.025
HOOS-QoL	33.0 ± 19.0	69.8 ± 22.7	33.2 ± 18.1	63.1 ± 24.7	32.2 ± 18.1	68.7 ± 22.8	.034

a Data are presented as mean ± SD. ADL, Activities of Daily Living; HOOS, Hip Disability and Osteoarthritis Outcome Score; iHOT-12, 12-item International Hip Outcome Tool; NAH, Non-Arthritic Hip; QoL, Quality of Life.

b *P* value is for the between-group difference in change from preoperatively to 2-year minimum follow-up (time × group interaction effect in 2-way ANOVA).

For the bilateral staged group, pre- to postoperative change in iHOT-12 score did not differ between those who had their bilateral procedures within 3 months of each other (38.5 ± 17.0 before surgery increasing to 67.2 ± 24.2 at follow-up) and those with a between-surgery interval >3 months (38.4 ± 18.5 presurgery; 77.1 ± 24.9 after surgery; *P* = .063).

The proportion of patients achieving the MCIC over all 3 groups ranged from 84.1% for the iHOT-12 score to 67.4% for HOOS-ADL. A lower proportion of patients receiving staged compared with simultaneous procedures achieved the MCIC for the iHOT-12 score (*P* = .038 for ANOVA; *P* = .032 for pairwise comparison) and HOOS-Sports (*P* = .020 and *P* = .015, respectively), and a lower proportion of those receiving staged compared with unilateral procedures achieved the MCIC for HOOS-QoL (*P* = .019 and *P* = .015, respectively) (Table 3).

**Table 3:** Proportion of Patients With Available PROM Scores Attaining MCIC^*a*^

	MCIC^*b*^	Simultaneous	Staged	Unilateral	*P*
iHOT-12 score	9.3	88.3	76.9	84.0	.038
NAH score	9.0	79.7	75.9	81.2	.391
HOOS-Symptoms	9.6	78.1	66.4	75.0	.063
HOOS-Pain	9.5	79.9	69.9	78.3	.095
HOOS-ADL	10.3	68.8	61.9	67.7	.412
HOOS-Sports	11.7	84.9	71.8	80.2	.020
HOOS-QoL	9.3	83.9	74.3	84.6	.019

a Data are presented in percentage. ADL, Activities of Daily Living; HOOS, Hip Disability and Osteoarthritis Outcome Score; iHOT-12, 12-item International Hip Outcome Tool; MCIC, minimal clinically important change; NAH, Non-Arthritic Hip; PROM, patient-reported outcome measure; QoL, Quality of Life.

b Calculated as 0.5 × preoperative standard deviation of outcome measure for all hip arthroscopies, according to the rationale and method of Norman et al.^21^

The overall revision/reoperation rate over a mean follow-up of 7.7 ± 3.2 years was 8.7% (34 hips) for the simultaneous bilateral group, 7.2% (16 hips) for the staged bilateral group, and 8.0% (122 hips) for the unilateral group (*P* = .81). Because follow-up duration was longer for simultaneous arthroscopies compared with staged and unilateral groups (*P* < .001) (Table 4), annualized rates (per 100 patients/year) were also compared, and these did not differ between groups (Table 4). In all, there were 4 (1.0%), 7 (3.2%), and 71 (4.6%) subsequent conversions to hip joint replacement in the simultaneous, staged, and unilateral groups, respectively (*P* = .003). However, comparison of annualized rates indicated no between-group differences in conversion to arthroplasty (Table 4). In terms of complications, there were 2 patients in the unilateral group who developed perineal numbness that resolved after 4 weeks. Both were men and had similar routine traction times. There were no such complications in the simultaneous and staged groups.

**Table 4:** Rate of Subsequent Revision/Reoperation and Conversion to Hip Arthroplasty on the Same Hip After Arthroscopic Procedures^*a*^

	Simultaneous (n = 392)	Staged (n = 222)	Unilateral (n = 1529)	*P*
Revision/reoperation, n	34	16	122	.81
Time to revision/reoperation, y	3.4 ± 2.2	3.9 ± 1.7	2.9 ± 1.9	.14
Follow-up duration, y	8.3 ± 2.4	7.6 ± 3.6	7.6 ± 3.2	<.001
Revision/reoperation rate, %/y	1.04	0.95	1.05	^*b*^
Hip joint replacement, n	4	7	71	.003
Time to hip joint replacement, y	3.7 ± 0.9	5.0 ± 4.3	3.4 ± 3.3	.48
Follow-up duration, y	8.0 ± 2.4	7.2 ± 3.6	7.3 ± 3.2	<.001
Hip joint replacement rate, %/y	0.13	0.44	0.64	^*c*^

a Data are presented as mean ± SD unless otherwise indicated.

b Pairwise 2-sample proportion test uncorrected *P* values as follows: simultaneous versus staged (*P* = .91), simultaneous versus unilateral (*P* > .99), and staged versus unilateral (*P* = .90).

c Pairwise 2-sample proportion test uncorrected *P* values: simultaneous versus staged (*P* = .45), simultaneous versus unilateral (*P* = .22), and staged versus unilateral (*P* = .72).

## Discussion

Our results show that patients who underwent simultaneous bilateral hip arthroscopy for FAI had similar outcomes to those receiving staged bilateral or unilateral surgery, with no additional risk of revision surgery or conversion to THA. Those having simultaneous surgery performed better than those receiving staged surgery in Sports- and QoL-specific indices of HOOS, having comparable outcomes to patients who underwent unilateral surgery. Similarly, a larger proportion of those receiving simultaneous arthroscopy achieved MCIC thresholds for these indices and for iHOT-12 total score. These results confirm those of smaller past studies.

Our original 2014 paper compared the PROMs (NAHS and Western Ontario and McMaster Universities Osteoarthritis Index), complication rate, postoperative pain, and time to return to daily activities between patients who underwent simultaneous bilateral (26 patients), staged bilateral (20 patients), and unilateral (33 patients) surgery. We found significant improvement in PROMs at the 1-year mark in all 3 groups, with no difference in complication rate, postoperative pain, and time to return to daily activities.^20^

Other encouraging papers have followed. McConkey et al^18^ studied a group of adolescent athletes comparing 12 patients who underwent simultaneous bilateral surgery with a matched control group of 12 patients who underwent unilateral surgery. Again, there was a significant improvement in the iHOT-12 scores over 2 years and no difference in the complication rates. Other groups have found comparable clinical outcomes between bilateral and unilateral surgeries^3,6,7,15^ and shown similar efficacy and comparable low complication rates and return to sports.^19,23,30^ In a systematic review including 6 studies, Fernandez et al^9^ reported that bilateral hip arthroscopy had similar efficacy to unilateral hip arthroscopy with no increased risk of complications. The minimum follow-up was 12 months for 3 studies and 24 months for the other 3 studies.

From our present single-surgeon cohort, we report results from 196 patients (392 hips) in the simultaneous group, almost 3 times the number in the systematic review by the Fernandez et al^9^, and with a minimum 2-year follow-up. This large sample provides sufficient statistical power to confirm that simultaneous bilateral surgery results in similar patient-reported outcomes to staged bilateral and unilateral surgery, and comparable low complication rates. Nonetheless, there were differences in selection between groups that are likely to have affected these outcomes, with those in the simultaneous group less likely than the staged group to have a nonzero Tönnis grade or severe acetabular chondral damage, or to require ≥5 microfracture holes. Another strength of this study is the use of the New Zealand Joint Registry data to track subsequent hip replacements for all patients over a mean of 7.4 years. Although it is a voluntary register, prospective entry of data into it is a mandatory requirement for members of the New Zealand Orthopaedic Association, and the register has a >96% capture rate of all public surgeries.^28^ Revision arthroscopy surgeries are rarely performed by other surgeons, and the capture rate of these is also likely to be very high due to the small community of surgeons performing hip arthroscopy in New Zealand and the relative experience of this senior surgeon compared with local peers.

Our data indicated that the staged bilateral group did not perform as well as other groups in certain PROM scores (HOOS-Sports, HOOS-QoL, and iHOT-12 score). Various selection factors might have contributed to this difference. One probable explanation is that this group was more likely to have been identified with advanced disease and counseled to have a staged procedure. A greater proportion had a nonzero Tönnis grade, had acetabular rim deterioration extending one-third or more into the socket, and required multiple (≥5) microfracture holes. Although the mean LCEA was similar between simultaneous and staged surgeries, a less stable socket was likely to be overrepresented in the staged group, with a higher proportion having borderline dysplasia and capsular laxity more likely. Other social or psychological factors may have been at play. For example, distracting pain or activity limitation from a severely affected hip may have meant that symptoms from a less symptomatic hip were not noticed or did not cause sufficient concern for simultaneous bilateral surgery to be chosen. Furthermore, some patients might not have been emotionally prepared for a bilateral surgery, or they may have been affected by anxiety or depression related to their symptoms. Patients’ home support structure and work requirements might also have influenced this decision.

Another factor that could explain the less favorable outcome in the staged compared with simultaneous group relates to the consequences of the interval between surgeries (median, approximately 2 months; mean, 3-4 months) on rehabilitation. There is emerging evidence that the timing of the contralateral surgery influences the overall outcome, although here we did not show a difference in pre- to postoperative iHOT-12 score changes between those having staged procedures ≤3 months apart, compared with those with a longer interval. The contralateral symptomatic hip can set back the rehabilitation of the index hip because the human body functions as a single system and “bilateral hip pathology should be considered as two halves of one problem, rather than as two separate entities,” as elegantly summed up by Owusu-Akyaw.^24^ Data surrounding the effect of intersurgery duration on outcomes in bilateral hip arthroscopy are inconclusive. Although 2 recent studies have shown reduced failure rates for bilateral surgeries staged less than a year apart,^29^ or <17 months apart,^7^ another showed no difference in PROMs between staged bilateral arthroscopy performed <3 months compared with >3 months apart.^3^ In the systematic review of bilateral versus unilateral hip arthroscopy, the mean interval between index and contralateral surgery in the staged bilateral group was 7.7 months.^9^

We recommend that patients be reviewed on case-by-case basis as we do not advocate simultaneous surgery if they have clinical or radiographic features that will likely warrant procedures requiring an extended period of postoperative protected weightbearing. These include treatment for cartilage damage (eg, microfracture) or for microinstability (ie, joint laxity or borderline hip dysplasia). Wheelchair ambulation can be highly inconvenient to patients and negates the advantages of faster rehabilitation. When considering simultaneous bilateral hip arthroscopy, there is also a need for an experienced surgeon and team. Matsuda et al^17^ shared their experience on how to improve surgical efficiency by using supine positioning and bilateral mobile leg spars, as well as reducing the traction time by taking off traction during acetabuloplasty. The option of a postless traction system might further reduce the complications related to prolonged traction time.^19^

### Limitations

There are several limitations in our study. First, there is an inherent bias in our selection of patients for simultaneous bilateral surgery. As this is not a prospective, randomized trial, the observed results cannot be generalized to any patient being treated for bilateral FAI. By excluding patients who might require procedures that entail a prolonged weightbearing restriction from simultaneous bilateral surgery, those with more complex and severe FAI will be directed to the staged bilateral group. This very likely contributed to better outcomes in the simultaneous group. The option of simultaneous surgery might also attract highly motivated patients who are more willing to rehabilitate well. While the design of the study is retrospective and nonrandomized, data collection is prospectively completed as part of an ongoing hip arthroscopy registry. A modest response rate for patient-reported outcomes may also introduce bias if those who did not complete preoperative or follow-up questionnaires had systematically different outcomes compared with those included in the analysis.

Another potential source of bias arises from evolution in the surgeon’s technique over the time frame of the study (June 2005 to December 2020) with the advent of new techniques, implants, and understanding of hip pathology. The longer mean follow-up time for the simultaneous group compared with other groups indicates earlier surgeries, on average, in this group. However, if anything, earlier surgery dates would be expected to impair outcomes in this group, underestimating the efficacy of the simultaneous procedure. All procedures were performed by a single high-volume surgeon who specializes in hip arthroscopic surgery. This could also limit the generalizability of the results.

Despite these limitations, we believe that this study, with larger numbers and longer follow-up, offers important reassurance that simultaneous bilateral arthroscopic FAI surgery is safe and effective. Hip arthroscopy is a relatively new procedure, and it is critical that this largest published cohort is followed over time to ensure that there are no unintended and unwanted consequences of simultaneous surgery.

## Conclusion

In a selected group of patients, simultaneous bilateral hip arthroscopy showed comparable surgical complication rates and patient-reported 2-year outcomes to staged bilateral and unilateral surgery. Simultaneous rather than staged bilateral surgery can reduce the overall rehabilitation period, allowing patients to return to their sports or work faster. This translates to economic savings in less time off work, and reduced health care costs with the single admission, anesthesia, surgery, and rehabilitation. The results from this study support the efficacy of simultaneous bilateral surgery for patients with appropriate indications.
